# Annotations

**Published:** 1904-03-19

**Authors:** 


### Annotations.

### Sanity and Sleep.

All the spiritual descendants of Sancho Panza, that finest eulogist of slumber, should bless the name of Dr. Selden Talcott, of New York. Dr. Talcott is a specialist in mental diseases, and he declares that the habit of early rising conduces to madness. "The free and lazy savage," he says, "gets up when he feels ready, and rarely or never becomes insane." Dr. Talcott's protest should be considered carefully by his fellow countrymen, who, even more than the British, pride themselves on their active habits. Recently, we believe, there has been founded in New York a society whose members pledge themselves not to take more than four hours' sleep in the twenty-four. It would be interesting to follow the health and life histories of those who keep this pledge. For we do not suppose that there is anything dangerous in early rising in itself, but only in the combination therewith of going to bed late. On the whole it is better to obey the old proverb and "go to bed with the lamb and get up with the lark." But it is a different thing when one combines the attempt to fulfil the latter part of the advice with a habit of going to bed with the nightingale. Yet the increasing strain of business compels early rising in those who would succeed, while if they want to combine pleasure with business they will find it impossible to get to bed before midnight or even then, for our amusements seem to begin and end later every year. The energetic people who go in for this fashion of life are apt to assure others, and themselves also, that they do not require much sleep, and undoubtedly it is possible with practice to do with much less than the normal amount of rest. It is important, however, to remember that such a habit cannot be indulged in without a corresponding sacrifice of health.

### Alopecia Areata.

In spite of the frequency with which alopecia areata is observed, its causation still remains a matter of uncertainty. Sabouraud's discovery of micro-organisms appeared at first to decide the issue in favour of the parasitic theory, but later observations have tended to question the occurrence of these in all cases, and to suggest that even when present they are not responsible for the development of the baldness. Mr. Jonathan Hutchinson continues to advocate his theory that alopecia areata is essentially connected with ringworm, and that the disease may develop many years after the attack of ringworm has been cured. He insists that alopecia is parasitic in origin, though he allows that the parasite has not been discovered. Mr. Hutchinson has recently published in the *Polyclinic* some further experiences in support of his teaching. On the other hand, it has frequently been remarked that alopecia is often associated with headache, neuralgias, and other signs of nerve disturbance, and recently Dr. Arthur Whitfield has suggested that reflex irritation resulting from eye strain may in certain cases be the immediate cause of the baldness. Dr. Whitfield disclaims the idea that he is advancing eye strain as a universal or even a very frequent cause, but he quotes certain experiences which are remarkable, and which ought to be borne in mind by those who may be called upon to treat this often intractable disorder. The significance of his observations extends obviously to the general doctrine of the ætiology of alopecia, and lends distinct support to those who insist that the disease is a tropho-neurosis. On this point it is worth considering whether the quarrel is not due to the fact that the rival disputants contemplate opposite sides of the shield. There certainly are cases which arise in circumstances that suggest contagion, and therefore parasitic influence, in the strongest possible manner. On the other hand, there are many examples of the disease in which no such suggestion arises, and in which depressed general health with other indications of defective nerve tone indicate that the alopecia is neurotic in origin. May it not be the case that in the two groups, though there is similarity in appearance, there is wide dissimilarity in the underlying cause? The truth of one of the contending theories in some cases does not exclude the accuracy of the rival theory in regard to others.

### Property and Life.

Long ago a newspaper declared that certain rebels must be taught "the value of life and the sacredness of property." The lay mind would incline to put life on a higher level than property, but it is doubtful if the judicial mind always does so. Certainly any cruelty that stops short of murder seems by many justices to be treated with more leniency than the smallest theft, or even a piece of truancy. One of our contemporaries quotes some examples of this which are sufficiently amusing—or would be if it were not such a serious matter. Thus, an apprentice fisherman, charged with "absenting himself from his employment" was sentenced to a month's imprisonment. A school girl, found guilty of having stolen threepence from a companion's pocket, was sentenced to fourteen days' imprisonment. This was exactly the length of imprisonment which the magistrates of Horsham thought a sufficient punishment for a father who had made two brutal assults on his seven year old son—on one occasion thrashing him with a razor-strop till great weals were raised all over his body, and on another striking him with a chair and causing a wound that had to be stitched and dressed by a surgeon. This offender, however, was given the option of a 15s. fine. No such option, however, did these same magistrates give to the man who stole three shirts—probably not new ones—from a clothes-line. He had to go to gaol for a month. Even he, however, got off better than the hapless Scot who was sentenced to six months' imprisonment for stealing a pair of boots from a shop. A fine certainly was inflicted on the poacher who was found with a hare in his possession—or in default of payment two months' imprisonment—but the fine was £5 in addition to the costs of the prosecution. Five pounds and costs, or two months' imprisonment, for stealing a hare—15s., or two weeks' imprisonment, for brutality to a child! Can anything show more clearly the superior importance of hares to the magisterial mind? Yet it is probable that the only case in which a long period of imprisonment would have done much good was just that of the unnatural father. A long sentence to him would at least have given his child a respite.

